# The eyespot resistance genes *Pch1* and *Pch2* of wheat are not homoeoloci

**DOI:** 10.1007/s00122-016-2796-x

**Published:** 2016-09-24

**Authors:** M. Pasquariello, J. Ham, C. Burt, J. Jahier, S. Paillard, C. Uauy, P. Nicholson

**Affiliations:** 1 John Innes Centre, Norwich Research Park, Colney, Norwich, NR4 7UH UK; 2 RAGT Seeds, Grange Road, Ickleton, Essex, CB10 1TA UK; 3 IGEPP, Institute for Genetics, Environment and Plant Protection, INRA, La Motte au Vicomte, 35650 Le Rheu, France

## Abstract

**Key message:**

**Phenotyping and mapping data reveal that chromosome intervals containing eyespot resistance genes** ***Pch1*** **and** ***Pch2*** **on 7D and 7A, respectively, do not overlap, and thus, these genes are not homoeloci.**

**Abstract:**

Eyespot is a stem-base fungal disease of cereals growing in temperate regions. Two main resistances are currently available for use in wheat. *Pch1* is a potent single major gene transferred to wheat from *Aegilops ventricosa* and located on the distal end of chromosome 7D. *Pch2*, a moderate resistance deriving from Cappelle Desprez, is located at the end of 7AL. The relative positions of *Pch1* and *Pch2* on 7D and 7A, respectively, suggest that they are homoeoloci. A single seed decent recombinant F7 population was used to refine the position of *Pch2* on 7A. New markers designed to 7D also allowed the position of *Pch1* to be further defined. We exploited the syntenic relationship between *Brachypodium distachyon* and wheat to develop 7A and 7D specific KASP markers tagging inter-varietal and interspecific SNPs and allow the comparison of the relative positions of *Pch1* and *Pch2* on 7D and 7A. Together, phenotyping and mapping data reveal that the intervals containing *Pch1* and *Pch2* do not overlap, and thus, they cannot be considered homoeloci. Using this information, we analysed two durum wheat lines carrying *Pch1* on 7A to determine whether the *Ae.ventricosa* introgression extended into the region associated with *Pch2*. This identified that the introgression is distal to *Pch2* on 7A, providing further evidence that the genes are not homoeoloci. However, it is feasible to use this material to pyramid *Pch1* and *Pch2* on 7A in a tetraploid background and also to increase the copy number of *Pch1* in combination with *Pch2* in a hexaploid background.

## Introduction

Eyespot is an important fungal disease of the stem base of cereals growing in temperate regions, such as North West Europe, North West USA, and New Zealand. It is caused by two species of fungi, *Oculimacula acuformis* and *O. yallundae* (Crous et al. 2003), and affects a wide range of hosts, including wheat, barley, rye, and oats. In the cases of severe infection, this causes lodging and premature ripening of grain, leading to reduced crop yield (Fitt et al. 1988). Two main sources of resistance effective at the seedling stage are currently available for use in wheat (*Pch1* and *Pch2*). The more potent of these is the single major gene *Pch1* which was transferred to hexaploid wheat from an accession (termed Vent 10) of the wild relative *Aegilops ventricosa* Tausch (2*n* = 4× = 28, genomes D^v^D^v^M^v^M^v^) and is located on the distal end of chromosome 7D (Doussinault et al. 1983; Maia 1967). *Pch1* has been mapped to a 0.26 cM region between five co-segregating proximal markers (*Xcos7*-*6*, *X4CD7A8*, *Xorw5*, *Xtr383*, and *Xwg7S*) and the distal COS (Conserved Orthologous Sequence) marker *Xcos7*-*9* (Burt and Nicholson 2011). Until recently, marker-assisted selection for *Pch1* commonly relied upon the presence of allele *Ep*-*D1b* of a co-segregating endopeptidase (McMillin et al. 1986). This assay has been replaced by the PCR-based STS (Sequence Tagged Site) marker *Xorw1* developed by Leonard et al. (2008).

It has been speculated that *Pch1* resistance may be conferred by the Ep-D1b protein (Worland et al. 1988) because of the apparent lack of recombination between *Xorw1*and *Pch1*. However, Mena et al. (1992) identified a recombination event between *Ep*-*D1b* and *Pch1* in a line that carried the endopeptidase allele *Ep*-*D1b*, but was susceptible to eyespot, suggesting that the resistance was not a product of the *Ep*-*D* locus. Eyespot resistance was also transferred to durum wheat from a second *Ae. ventricosa* accession (Vent 11) (Huguet-Robert et al. 2001). This accession lacks a functional Ep-D1 endopeptidase allele further indicating that *Pch1* is not a product of *Ep*-*D1b*.

*Pch1* has been widely used in attempts to increase eyespot resistance, but its presence in commercial wheat varieties in Europe has been limited because of linkage drag with undesirable genes derived from *Ae. ventricosa*. Although *Pch1* is highly effective against both eyespot species, a significant reduction of yield and thousand-kernel mass has been sometimes observed in the absence of the disease (Koen et al. 2002). Moreover, even though recombination does occur between the *Ae. ventricosa* 7D^v^ and the wheat 7D chromosome segments, it does so at a much lower frequency than normal (Worland et al. 1988), and until now, it has been difficult to break the linkage between *Pch1* and yield-limiting traits.

The second source of eyespot resistance derives from the French cultivar Cappelle Desprez (CD) (Vincent et al. 1952). Since the 1950s, this has proved to be a moderate but highly durable resistance, dominating European wheat markets for over two decades. Originally, most of the CD resistance effects were determined to be conferred by a seedling resistance locus termed *Pch*2, located on the distal end of chromosome 7AL (de la Peña et al. 1996), but more recently, an additional QTL of moderate effect has been located on chromosome 5A (Burt et al. 2011; Muranty et al. 2002). *Pch2* has been mapped to a 7 cM interval between SSR markers *Xwmc346* and *Xcfa2040*, and it is closely associated with SSR *Xwmc525* (Chapman et al. 2008).

The relative positions of *Pch1* and *Pch2* on the long arm of chromosomes 7D and 7A, respectively, suggest that they are homoeoloci. A number of the previous studies support this hypothesis. For example, in the work of Chapman et al. (2008), the SSR marker *Xcfa2040*, which has homoeoloci on 7A and 7D, was found to be close to *Pch2* on 7A, and also to the *Pch1*-linked SSR marker *Xwmc14* on 7D. Unfortunately, due to the homoeologue-specific nature of most SSR markers, it was impossible to make other direct comparisons between the two regions. The potential homoeology is further supported by the study of de la Peña et al. (1997), where they mapped *Ep*-*D1* and *Ep*-*A1*, respectively, on the distal portion of the long arms of the 7D and 7A chromosomes. However, Leonard et al. (2008), who identified a candidate gene for Ep-D1 and developed a DNA-based marker on it, reported a large discrepancy between the genetic distances of the two endopeptidase loci (*Ep*-*D1* and *Ep*-*A1*) and the *Pch* loci on their respective chromosomes and could not provide clear evidence to support them being homoeologous based upon their genetic positions. More recently, Burt and Nicholson (2011) found that the region containing *Pch1* (flanked by the markers *Xcos7*-*6* and *Xcos7*-*9*) on chromosome 7D overlapped with the *Pch2* QTL between markers *Xcos7*-*6* and *Xorw1*. The *Pch2* QTL region originally spanned a 7 cM region (Chapman et al. 2008), but the addition of COS and sequence tagged site (STS) markers extended both the map and the QTL interval (Burt and Nicholson 2011). The large size of the QTL interval was believed to be, largely, a result of inaccuracies in phenotyping F3 families for eyespot resistance conferred by *Pch2*, as this gene is only of moderate effect (Burt et al. 2010; Chapman et al. 2008).

A high level of genome-synteny between wheat and model species, such as rice (*Oryza sativa*) sorghum (*Sorghum bicolor*) and Brachypodium (*Brachypodium distachyon*), has been widely reported (Vogel et al. 2010), with Brachypodium providing a greater level of co-linearity with wheat than the other two due to its closer evolutionary relationship. Notably, the panel of co-dominant COS markers developed by Burt and Nicholson (2011) was obtained by exploiting the syntenic relationship between mainly Brachypodium (but also rice and sorghum) and wheat. The availability of complete genomic sequence of these species is a great advantage for the development of molecular markers, for the identification of candidate genes for traits of interest and for predicting biological gene functions. Such comparative and functional genomic approaches have already helped to uncover the molecular basis of important wheat characters, including disease resistance (Krattinger et al. 2009), vernalisation requirement (Yan et al. 2006), and flowering time (Lv et al. 2014). To aid the genetic dissection of eyespot resistance, this co-linear relationship could be further explored for proceeding to the map-based cloning of *Pch1* and at the same time, it could be helpful for developing homoeologue (7A/7D) transferable markers by which to resolve the potential homoeology between *Pch1* and *Pch2*.

Although there are limitations in utilising the *Ae. ventricosa* introgression, it is being actively used both in Europe and in the USA by wheat breeders. However, considering the fact that *Pch1*-carrying varieties can be affected by significant eyespot-induced grain yield losses in situations of severe attack, there is both an interest and a need to further enhance the resistance of wheat. A first strategy to achieve this goal is through pyramiding of *Pch1* and the *Pch2* and 5A QTL of Cappelle Desprez. Pyramiding entails stacking multiple resistance genes or QTLs in a variety to develop stronger and durable resistance. A first attempt in this direction for eyespot was reported by Hollins et al. (1988) who stated that the variety Rendezvous, derived from crosses involving VPM ([*Ae.* ***v*** *entricosa* × *T.* ***p*** *ersicum*] × **M**arne) and CD derived lines, displays higher resistance than VPM. Consequently, they suggested that Rendezvous possesses resistance from both *Ae. ventricosa* and CD, but at that time, it was not possible to confirm this, as suitable molecular markers were not available for *Pch2*. Later, Burt et al. (2010), using SSR markers, identified some additional wheat lines containing both *Pch1* and *Pch2*, such as the intergenotypic single chromosome substitution lines Hobbit ‘sib’-VPM7D (HS⁄VPM7D) and the variety Lynx.

A second strategy to improve eyespot resistance in wheat is the generation of genotypes carrying additional copies of *Pch1* on either 7A and/or 7B chromosomes as well as 7D. Stacking multiple *Pch1* copies in the genome to improve the level of resistance compared to a single copy is based on the observation that plants homozygous for *Pch1* are more resistant to eyespot than those that are heterozygous (Huguet-Robert et al. 2001). As a first step towards this objective, Huguet-Robert et al. (2001) produced meiotically stable *Pch1*-carrying tetraploid wheat lines derived from backcross progenies of a cross between *Ae. ventricosa* and *T. durum* cv. Creso *ph1c*. The *Pch1* transfer, resulting from recombination between homeologous chromosomes, was located on the long arm of chromosome 7A. This conclusion was based on the inability to detect the 7A specific SSR marker *Xgwm698* or the Ep-A1 protein in the homozygous recombinant lines, suggesting that a recombination event had occurred between the 7A and 7D^*v*^ chromosomes.

The four goals of the present study were to: (1) develop new gene-based molecular markers closely linked to *Pch1* and *Pch2* by exploiting synteny between wheat and *Brachypodium distachyon*; (2) refine the genetic position of *Pch1* and *Pch2*, respectively, on the 7D and 7A chromosomes; (3) clarify the potential homoeologous relationship between *Pch1* and *Pch2*; (4) characterize the *T. durum* wheat lines carrying *Pch1* on the 7A chromosome created by Huguet-Robert et al. (2001) using SSRs and new developed KASP markers.

## Materials and methods

### Plant material

Nine hundred and forty-four BC_6_ F_2_ plants from the cross between the recombinant substitution line RVPM25 and Hobbit-Sib (HS) produced by Burt and Nicholson (2011) were genotyped to identify additional recombinants in the *Pch1* region. The 25 BC_6_ F_2_ plants reported by Burt and Nicholson (2011) to be recombinant between the SSR markers *Xbarc76* and *Xcfd175* were re-analysed with additional markers to refine the recombination break points.

A total of 92 F_7_ lines obtained by single seed decent (SSD) and deriving from a cross between Chinese Spring (CS) and the substitution line Chinese Spring/Cappelle Desprez 7A (CS/CD7A) produced by Chapman et al. (2008) were used to define the genetic position of *Pch2*.

The *T. durum* line 301 produced by Huguet-Robert et al. (2001) and containing the *Pch1* locus from *Ae. ventricosa* (Vent11) on chromosome 7A was crossed to Creso *ph1c* as described previously Huguet-Robert et al. (2001) to reduce further the size of the *Ae. ventricosa* introgression. Two lines, Red1 and Red5, were selected for further analysis based upon the presence of the 7A-specific SSR marker *Xgwm698* in these lines (data not shown). Red1 and Red5 were characterized using the sets of markers for *Pch1* and *Pch2* developed within the present study along with a panel of 7A and 7D specific SSRs to better define the size of the *Ae. ventricosa* introgression in each line.

### Seedling bioassays

The 92 CS × CS/CD7A F_7_ lines were tested for eyespot resistance against *O. acuformis* at the seedling stage. Five plants from each of the F_7_ lines were grown in 7 × 7 cm square pots containing peat and sand compost. Three replicate pots of each, with five plants per pot, were arranged in a complete randomized block design. The parental lines, CS and CS/CD7A, were included in each block as a control. The plants were grown for 2–3 weeks in a controlled-environment room (CER) at 8 °C and 8/16 h light/dark condition, then inoculated using inoculum slurry constrained within PVC cylinders, and incubated as described by Chapman et al. (2008). Plants were harvested 8 weeks after inoculation and scored for disease using the method described by Scott (1971). This experiment was subsequently repeated using identical methods to confirm the findings.

For the eyespot inoculation, a homogenized mixture of 25 isolates of *O. acuformis* was selected from the JIC culture collection. A mixture of different isolates was used to ensure that a successful infection was achieved in the case of lack of virulence of one or more isolates. Each isolate was grown on V8 agar (9 g of bacto-agar and 50 ml of V8 vegetable juice in 450 ml of deionised water) at 15 °C for 21 days prior to preparation of inoculum as described previously (Chapman et al. 2008).

### Statistical analysis

For the two CER experiments, the analysis of variance was performed on visual disease scores to assess the variation attributable to line, blocks, and interactions between line and blocks, using a general linear model (GLM) in Genstat v.16 (Copyright 2009 Lawes Agricultural Trust, Rothamsted Experimental Station, UK). Predicted mean disease scores were calculated for each line using the GLM for use in the QTL analysis.

### Map construction and QTL analysis

Genetic maps were generated for *Pch1* and *Pch2* populations in JoinMap© (version 3.0) (Stam 1993) using default parameters. The *Pch2* linkage map data were combined with phenotypic data from the two seedling bioassays for QTL analysis. The QTL analysis was carried out using data from each phenotype trial individually as well as using a data set in which the data from the two trials were combined.

The search for QTLs was done using the Single Trait Linkage Analysis of Genstat v.16 (Copyright 2009 Lawes Agricultural Trust, Rothamsted Experimental Station, UK) in three different steps: (1) initial genome-wide scan by simple interval mapping (SIM) to obtain candidate QTL positions; (2) one or more rounds of composite interval mapping (CIM), in the presence of cofactors, which are potential QTL positions detected at the previous step; and (3) fit the final QTL model. Default threshold based on the estimation of the effective number of tests (Li and Ji 2005) has been chosen for the QTL analysis.

### Molecular markers

The mapping reported by Burt and Nicholson (2011) located *Pch1* to the region between the markers *Xcos7*-*6* (corresponding to *Bradi1g29690*) and *Xcos7*-*9* (corresponding to *Bradi1g29287*). To refine the position of *Pch1* on the chromosome 7D, markers were developed taking advantage of the syntenic relationship between *Brachypodium distachyon* (Bd) chromosome 1 and wheat Group 7 chromosomes. The *Pch1* homologous location on Bd chromosome 1 was identified in EnsemblPlants (http://plants.ensembl.org/Brachypodium_distachyon/Info/Index) and found to cover approximately a region of 424 Kb (Bd1:24810968-25235129) containing 43 genes from *Bradi1g29690* to *Bradi1g29287*.

Wheat genes on the group 7 chromosomes corresponding to each of these Bd genes were identified, and PCR primer pairs were designed on 23 of them at locations with high levels of sequence conservation between Bd and wheat to maximise the probability of obtaining successful amplification of the *Ae. ventricosa* introgressed DNA. These primers were tested on genomic DNA from HS and RVPM25, and PCR products were used in two different strategies for producing either Single-Strand Conformation Polymorphism (SSCP) or Kompetitive Allele Specific PCR (KASP) markers. In the first case, PCR products were examined by SSCP assay (Martins-Lopes et al. 2001) using Sequa Gel^®^ MD (National Diagnostics, UK Ltd.) and visualised by silver staining (Bassam et al. 1991). For KASPs, instead, PCR products were sequenced and aligned to identify SNPs between *Ae. ventricosa* and wheat 7D. KASP marker PCR assays were manually designed on the basis of the identified SNPs.

A similar procedure was followed for identifying new markers to define the *Pch2* locus and to allow the genetic maps for 7A and 7D to be related to one another. *Pch2* was originally located on the chromosome 7A to a position between the markers *Xwmc346* (SSR) and *Xorw1* (corresponding to *Bradi1g29400*) (Burt and Nicholson 2011). Due to the non-gene-based nature of SSR markers, it was impossible to identify a syntenic position for *Xwmc346* on Bd1. Therefore, to ensure the entire coverage of the potential homologous region of *Pch2* on Brachypodium, a large Bd1 region containing about 100 genes was identified. As one of the aims of this work is to determine the homoeologous relationship between *Pch1* and *Pch2*, most attention was focused on developing markers to the overlapping segment of the two loci. Eight Bd genes were selected at equal intervals to span the region from *Bradi1g29480* at the proximal end of *Pch1* to *Bradi1g29960* at the distal end of *Pch2*.

Initially, primer pairs were designed in the most conserved region and tested on CS and CS/CD7A. Sequencing of PCR products then allowed the identification of 7A homoeologue-specific SNPs on the basis of which KASP assays were designed.

A second set of KASP markers specific for the *Pch2* map was developed using the high-density single nucleotide polymorphism (SNP) genotyping iSelect array of 81,587 SNPs published by Wang et al. (2014) and available for 400 wheat lines on the CerealDB website (http://www.cerealsdb.uk.net/cerealgenomics/CerealsDB/iselect_search.php). Both Chinese Spring (CS) and Cappelle Desprez were included among this panel. After analysing the complete set of SNPs mapping to 7A and polymorphic between the two parental lines CS and Cappelle Desprez, a set of SNPs located across the *Pch2* region interval was selected. KASP primers were designed using PolyMarker, an automated bioinformatics pipeline for SNP assay development which is designed to increase the probability of generating homoeologue-specific assays for polyploid wheat [http://polymarker.tgac.ac.uk/ (Ramirez-Gonzalez et al. 2015)].

Thermodynamic properties of designed primers were verified after adding the standard FAM or HEX compatible tails (LGC ltd).

7A and 7D specific KASP markers were initially tested against the parental lines (CS and CS/CD7A, HS, and RVPM25, respectively), and the ones which were polymorphic between the parental line pairs were then applied to the F_7_ CSxCS/CD7A and BC6 F_2_ HSxRVPM25 populations, respectively.

Genomic DNA extraction from the parental lines was performed using the CTAB method (Nicholson et al. 1996) to obtain large quantities of high-quality nucleic acid, whereas genomic DNA of the two population lines was extracted using the extraction protocol for 96-well plates, adapted from Pallotta et al. (2003). PCR reactions were prepared in a 6.25 µl final volume containing 2.5 µl DNA (10 ng/µl), 3.125 µl Taq mastermix (Qiagen), and 0.625 µl of the relevant primer pair (2 µM). A common PCR programme was used throughout consisting of a denaturing step of 95 °C for 5 min, followed by 35 cycles of 95 °C for 30 s, 58 °C for 30 s, and 72 °C for 1 min, with a final elongation step of 72 °C for 7 min. Where required, PCR products were then purified using the QIAquick Gel Extraction Kit (Qiagen), sequenced using BigDye^®^ Terminator v3.1 Cycle Sequencing Kit (following the manufacturer’s instructions), and aligned in VectorNTI^®^ (ThermoScientific).

For the KASP assay, 2 µl (5 ng/µl) of the extracted DNA were added to 0.056 µl of primer mix (12 µl each of specific primer, 30 µl of the common primer, and 46 µl deionized water) and 2 µl of KASP master mix (LGC). The PCR included an initial denaturation step of 94 °C for 15 min followed by 10 cycles of touchdown PCR (annealing at 62–56.6 °C and decreasing at 0.6 °C per cycle), then 25 cycles of 94 °C for 10 s and 60 °C for 1 min. After amplification, plates were read on a Tecan Safire plate reader and genotyped using the Klustercaller™ software (version 2.22.0.5, LGC).

A set of publicly available SSR markers was used to test the two *T. durum* lines Red1 and Red5 produced by Huguet-Robert et al. (2001). Markers were chosen to cover the 7A and 7D regions, where KASPs were not predictive. Primer sets were selected from the Wheat Microsatellite Consortium, as described within ‘GrainGenes’ (http://wheat.pw.usda.gov/GG2/index.shtml). PCR conditions for SSR amplification were as described previously with annealing temperatures as indicated by the ‘GrainGenes’ website. Samples were prepared by adding 1 µl of a 1:5 dilution of the PCR product to 10 µl formamide and 0.2 µl of LIZ 500 size standard (Applied Biosystems). Samples were run on an ABI 3700 capillary sequencer (Applied Biosystems), and the output data were analysed using Peak Scanner v1.0 (Applied Biosystems) to determine the product size of the amplicons.

## Results

### Refining the position of *Pch1* on chromosome 7D

To refine the position of *Pch1* on chromosome 7D, the available set of six COS and SSR markers (*Xtr331*, *Xtr370*, *Xwg7s*, *Xorw5*, *Xbarc76*, and *Xcfd175*) previously produced by Burt and Nicholson (2011) was supplemented by a set of 11 new markers, three SSCPs (*X29550*, *X29570*, and *X29577*) and eight KASPs (*Xorw1_D*, *Xcos7*-*11_D*, *Xcos7*-*9_D*, *Xtr40_D*, *X29457*, *X29500*, *X29515*, and *X29560*) developed utilising the syntenic relationship between wheat and Bd. They were named using an “*X*” followed by a five digit number indicating the syntenic Bd gene and are listed in Table 1 along with primer sequences, the relevant syntenic Bd gene number, and the corresponding 7D wheat gene. Different names were used for *Xorw1_D*, *Xcos7*-*11_D*, *Xcos7*-*9_D*, and *Xtr40_D* to clearly indicate that new KASP markers were developed on the same genes previously described and mapped by Burt and Nicholson (2011). A set of 23 7D located wheat genes covering the *Pch1* homologous location on Brachypodium chromosome 1 was used to design molecular markers, but only 11 of them were useful for development of 7D/ventricosa specific KASPs or SSCPs.

**Table 1:** Summary of markers used to identify recombinants in the region of *Pch1*

Marker name	Brachypodium gene	Wheat 7D homolog	Marker TYPE	Primers sequence
*X29577*	*Bradi1g29577*	Traes_7DL_C255A109C	SSCP	tccacttgaggttgtcaaga
				attgcaagctctaggaaaca
*X29570*	*Bradi1g29570*	Traes_7DL_471B4134B	SSCP	caccgttgttttcactgctg
				gtccctagaataggggagca
*X29560*	*Bradi1g29560*	Traes_7DL_6F30AD134	KASP	ggatgacacatgatctCctacgA
				ggatgacacatgatctCctacgC
				atctgttatcagGaagcgggcA
*X29550*	*Bradi1g29550*	**–**	SSCP	ccaactcgcacctcatcac
				cgttgatctcgtccacgtc
*X29515*	*Bradi1g29515*	Traes_7DL_0B4336C25	KASP	ctttcttctctcgcatgtgaacatA
				ctttcttctctcgcatgtgaacatG
				atttggtggaccttctgtcactG
*X29500*	*Bradi1g29500*	Traes_7DL_35DFF1FCF	KASP	cttattggattggatcagtttggcA
				cttattggattggatcagtttagcT
				ctcaaagtcctcaactatctggaaa
*X29457*	*Bradi1g29457*	Traes_7DL_B2EB69EC9	KASP	agaccttgtatttggtgcagcG
				agaccttgtatttggtgcagcA
				ttaggtgtgggctctaggtttct
*Xorw1_D*	*Bradi1g29400*	Traes_7DL_DBBA5FD6C	KASP	ctcatatccattgtgcttggttGcC
				ctcatatccattgtgcttggttAcT
				ccctctatctttgactatgacatgg
*Xcos7*-*11_D*	*Bradi1g29441*	Traes_7DL_973A33763	KASP	tattgatgcagaaacaccctTcgcT
				tattgatgcagaaacaccctAcgcA
				aattcctcgtcgtcgtcaccg
*Xtr40_D*	*Bradi1g29320*	Traes_7DL_434E0F3E1	KASP	tgtcatatactctccagcagaagaAA
				tgtcatatactctccagcagaagaGG
				gacataagtgcataggtgcttgtg
*Xcos7*-*9_D*	*Bradi1g29287*	Traes_7DL_CA193C813	KASP	ccctagctgaccgCcC
				ccctagctgaccgGcT
				caccgccgtggcatt

Two main reasons account for this low successful ratio (48 %): (1) PCR primers designed at locations with high levels of sequence conservation between Bd and wheat failed to amplify a specific PCR product on the *Ae. ventricosa* introgressed DNA (seven primer pairs; 30 %) and (2) after sequencing of PCR products, no useful SNPs between *Ae. ventricosa* and wheat 7D genes were identified (five primer pairs; 22 %).

**Fig. 1:**
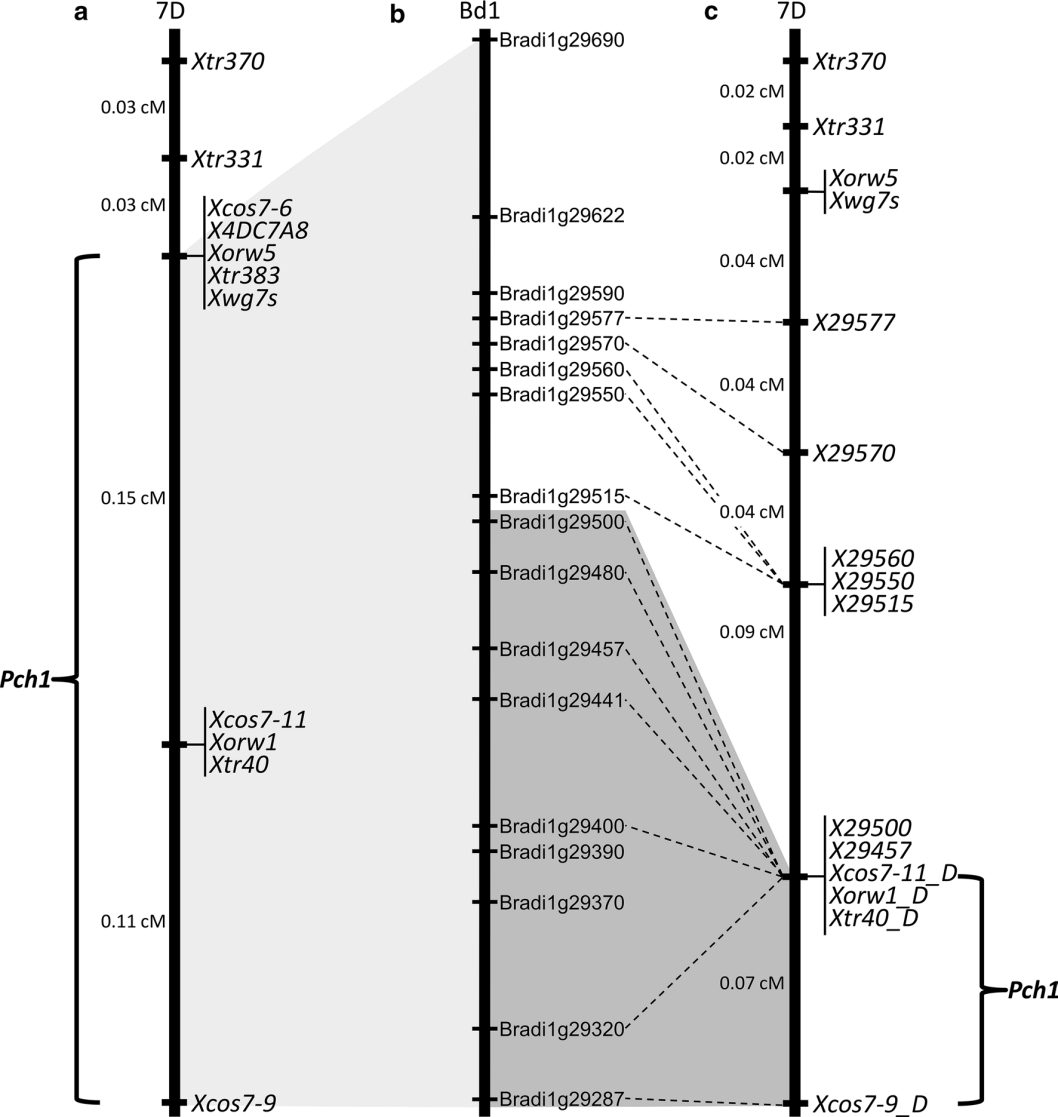
Comparison of the wheat chromosome 7D genetic map, including *Pch1*, published by Burt and Nicholson (2011) and the new version of the same map using physical marker locations on Brachypodium chromosome 1 as a reference. **a** *Pch1* genetic map published by Burt and Nicholson (2011). **b** Gene-spaced map of Brachypodium chromosome 1. **c** New genetic map of *Pch1*

These markers were applied to 2256 lines of the HS x RVPM25 BC_6_ F_2_ population [the original 1312 lines reported by Burt and Nicholson (2011) plus an additional 944 lines]. Six BC_6_ F_2_ plants from the 2256 tested (0.26 %) were identified with recombination events occurring between the KASP markers *X29577* (corresponding to *Bradi1g29577*) and *Xcos7*-*9_D* (corresponding to *Bradi1g29287*). Two groups of co-segregating markers, *X29560*-*X29550*-*X29515* and *X29500*-*X29457*-*Xcos7*-*11_D*-*Xorw1_D*-*Xtr40_D*, were identified within the six plants. This probably reflects the very low recombination rate in the region, and suggests that they sit very close to or within the *Ae. ventricosa* introgressed segment, where the recombination is inhibited or hindered. The available recombination events were used to determine the marker order in the *Pch1* region, and a new genetic map was constructed, as shown in Fig. 1. In the new version of the map, *Pch1* spans a genetic region of 0.07 cM from the distal marker *Xcos7*-*9_D* (*Bradi1g29287*) to the proximal group of co-segregating markers *X29500*-*X29457*-*Xcos7*-*11_D*-*Xorw1_D*-*Xtr40_D*. The refined position of *Pch1* on the chromosome 7D eliminates 19 genes of the syntenic region on Bd1 from the list of candidate genes in comparison with the previous version of the map published by Burt and Nicholson (2011). As a result, the *Pch1* syntenic region of Bd1 is reduced from 424 to 250 Kb long and includes 24 genes (Fig. 1).

### *Pch2* mapping and QTL analysis

A new set of 21 KASP markers was used to refine the position of *Pch2* on chromosome 7A. 11 of these (*X29960*, *X29947*, *X29792*, *X29776*, *X29560*, *X29500*, *X29480*, *Xcos7*-*11_A*, *Xorw1_A*, *Xtr40_A*, and *X29390*) are gene-based markers and were developed taking advantage of the syntenic relationship between Bd and wheat. In particular, *X29960*, *X29947*, *X29792*, *X29776*, *X29560*, *X29500*, and *X29480* were designed on 7A wheat genes, which, based on the synteny with Brachypodium, were determined to be located in the overlapping segment of *Pch1* and *Pch2*. Moreover, *Xcos7*-*11_A*, *Xorw1_A*, and *Xtr40_A* are new gene-based KASP markers developed on the same genes previously described and mapped on the 7A map by Burt and Nicholson (2011).

The other ten markers (*XBS1_30210*, *XBS2*, *XBS3_29990*, *XBS4_29980*, *XBS5*, *XBS6*, *XBS7*, *XBS8_29590*, *XBS9_29370*, and *XBS10*) were developed from the high-density single nucleotide polymorphism (SNP) genotyping iSelect array (Wang et al. 2014) and are based on 7A specific SNPs able to distinguish between CS and CD. Only five of them could be related to wheat genes and their corresponding Bd gene code is indicated in their nomenclature (*XBS1_30210*, *XBS3_29990*, *XBS4_29980*, *XBS8_29590*, and *XBS9_29370*).

These two new sets of markers, the Bd-wheat based set (X) and the iSelect set (XBS), are listed in Table 2 along with primer sequences, corresponding Bd gene code (where available) and 7A wheat ESTs’ gene names where known.

**Table 2:** Summary of markers used to identify recombinants in the *Pch2* region

Marker name	Brachypodium gene	Wheat 7A homolog	BS SNP code	Marker type	Primer
*X29390*	*Bradi1g29390*	Traes_7AL_ACE7F3AC3	–	KASP	gtttgtgtagaacagaggaagcaT
					gtttgtgtagaacagaggaagcaG
					acacatcaatgtttgacttccg
*X29480*	*Bradi1g29480*	Traes_7AL_1522CB55B	–	KASP	gtcgtctagtgtttcttacttgC
					gtcgtctagtgtttcttacttgG
					gacgactgctgttgcctagacttat
*X29500*	*Bradi1g29500*	Traes_7AL_AFA2C29C9	–	KASP	ggatgttgatgagagctgagaggA
					ggatgttgatgagagctgagaggC
					atgggcaaatccagcataatgc
*X29560*	*Bradi1g29560*	Traes_7AL_3F2C16688	–	KASP	tcctctggctatcaaggtcatT
					tcctctggctatcaaggtcatC
					catcgctattcaggaccatctg
*X29776*	*Bradi1g29776*	Traes_7AL_13AE7EEC0	–	KASP	attctaggtgcagaagggaatcA
					attctaggtgcagaagggaatcG
					ggtctgctttgtcatcctgaaatc
*X29792*	*Bradi1g29792*	Traes_7AL_DA8343C6B	BS00072731	KASP	cctcttccatcggaaacctcA
					cctcttccatcggaaacctcG
					agtgctgaaagttttgtcaaattc
*X29947*	*Bradi1g29947*	Traes_7AL_842775A88	BS00043010	KASP	ctttgacagtgacattctgttcC
					ctttgacagtgacattctgttcA
					cctcatcagccatcaaatattagcT
*X29960*	*Bradi1g29960*	Traes_7AL_DF750E6B1	–	KASP	catgcacttgttgttctccatccG
					catgcacttgttgttctccatccA
					cagtttgataaccatgtgctcctcaa
*XBS1_30210*	*Bradi1g30210*	Traes_7AL_09A36AAE41	BS00117383	KASP	gctccgcttctaggccaT
					gctccgcttctaggccaG
					gtagtagtaacggcagagacaa
*XBS2*	*–*	–	BS00139278	KASP	gccttaccaaactacgcacG
					gccttaccaaactacgcacA
					attgtttcttcgtgcgatccg
*XBS3_29990*	*Bradi1g29990*	Traes_7AL_6ACB3177B	BS00014246	KASP	cgacgatgactcggacatctcA
					cgacgatgactcggacatctcG
					ggggcgcaaagataaaatcgtccat
*XBS4_29980*	*Bradi1g29980*	Traes_7AL_BB11D77B5	BS00023109	KASP	gaggcccatcatgcgcagcA
					gaggcccatcatgcgcagcA
					agaggcccgatgtcaagttggataa
*XBS5*	*–*	–	BS00177197	KASP	gaaggtcgccagtatatatgtgT
					gaaggtcgccagtatatatgtgC
					atttggcataaggaagcgcg
*XBS6*	*–*	–	BS00067682	KASP	ccaagcaatttcggtgcagttA
					ccaagcaatttcggtgcagttG
					gtagctggggaaactaatacaagtc
*XBS7*	*–*	–	BS00068032	KASP	cccaaaggacaacttagtgtcG
					cccaaaggacaacttagtgtcA
					caagacatacatgacgtgaggta
*XBS8_29590*	*Bradi1g29590*	Traes_7AL_5262BD5AE	BS00022137	KASP	taggctagatagaatgaaacatggcA
					taggctagatagaatgaaacatggcG
					ggatgcagagctccaaggcagat
*XBS9_29370*	*Bradi1g29370*	–	BS00023200	KASP	agagaactgatatgtcggcgaT
					agagaactgatatgtcggcgaC
					gaagaaactcgagctgctcaaggtt
*XBS10*	*–*	–	BS00022447	KASP	cacagatcacgcgggcgcaG
					cacagatcacgcgggcgcaA
					ggcgcaggtcgacccgcat
*Xcos7-11_A*	*Bradi1g29441*	Traes_7AL_04C7DF2DE		KASP	tcctgagagttctttgcattgaaG
					tcctgagagttctttgcattgaaA
					cctaccaattacactggagaagtcttt
*Xorw1_A*	*Bradi1g29400*	Traes_7AL_D1DB1B9EE		KASP	cgaattggttgcttgcccA
					cgaattggttgcttgcccG
					cgacaagactgaccttccag
*Xtr40_A*	*Bradi1g29320*	Traes_7AL_33AA9A079		KASP	aaatggccaaataacagcaacA
					aaatggccaaataacagcaacG
					tgtttccttccatgtagtctcc

**Fig. 2:**
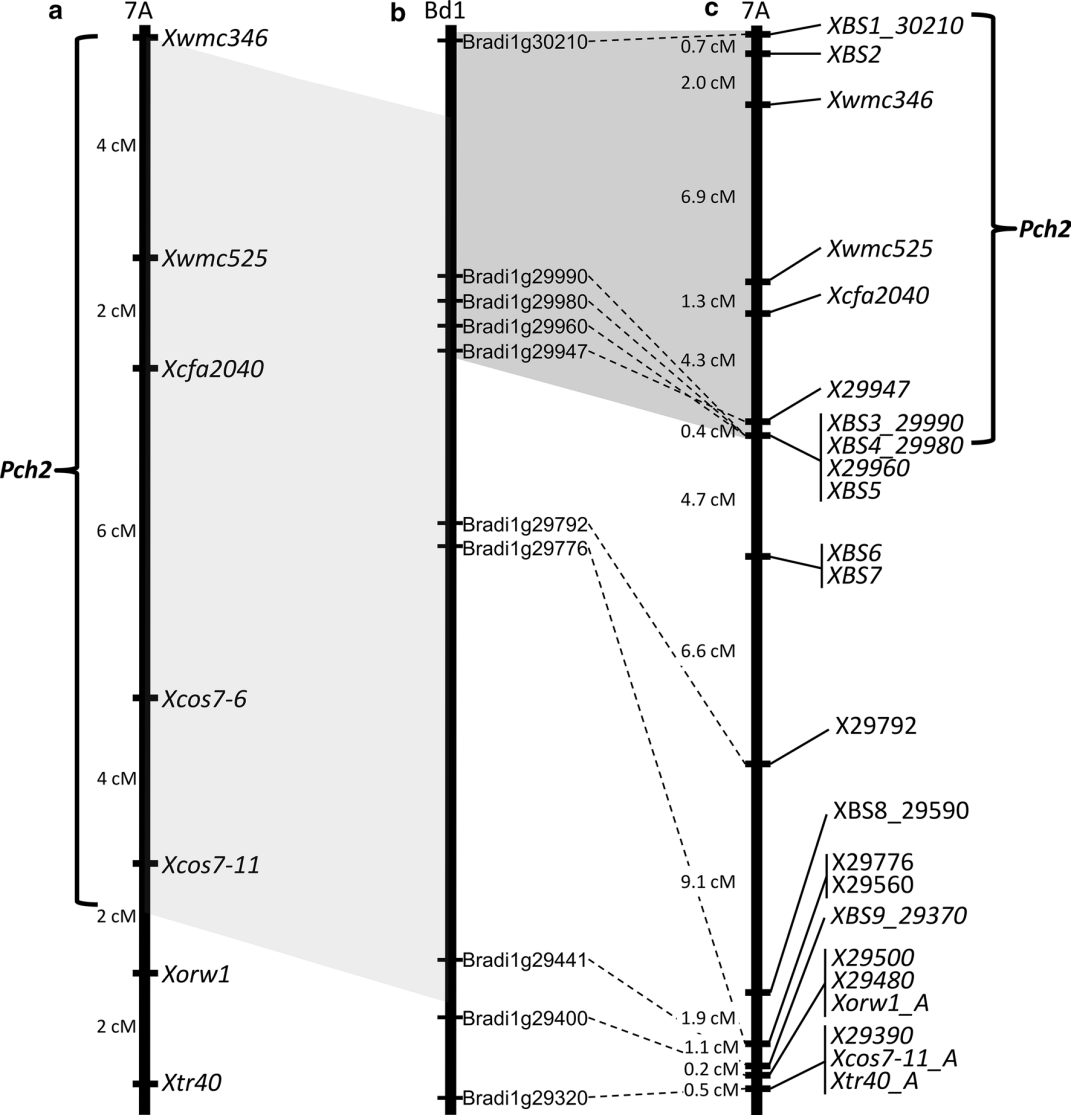
Comparison of the wheat chromosome 7A genetic map, including *Pch2*, published by Burt and Nicholson (2011) and the new version of the same map using physical marker locations on Brachypodium chromosome 1 as a reference. **a** *Pch2* genetic map published by Burt and Nicholson (2011). **b** Gene-spaced map of Brachypodium chromosome 1. **c** New genetic map of *Pch2*

These two sets of markers, together with the ones previously described by Burt and Nicholson (2011) (*Xwmc346*, *Xwmc525*, and *Xcfa2040*) were used to genotype 92 lines of the CS × CS/CD7A SSD F_7_ population, and a new genetic map of the region, spanning a total of 43 cM, was produced (Fig. 2).

Moreover, the same set of lines was phenotyped for eyespot resistance in two replicated and independent seedling disease bioassays inoculated with *O. acuformis*. It has been reported that *Pch2* is more effective against *O. acuformis* than against *O. yallundae* (Burt et al. 2010). Earlier attempts to localise *Pch2* were hindered because of the use of F_3_ families (still segregating for *Pch2*) rather than more advanced fixed lines for assessing disease resistance (Chapman et al. 2008, 2009). For these reasons, in the current work, disease assessment was carried out using an SSD F_7_ population challenged with isolates of *O. acuformis*.

**Fig. 3:**
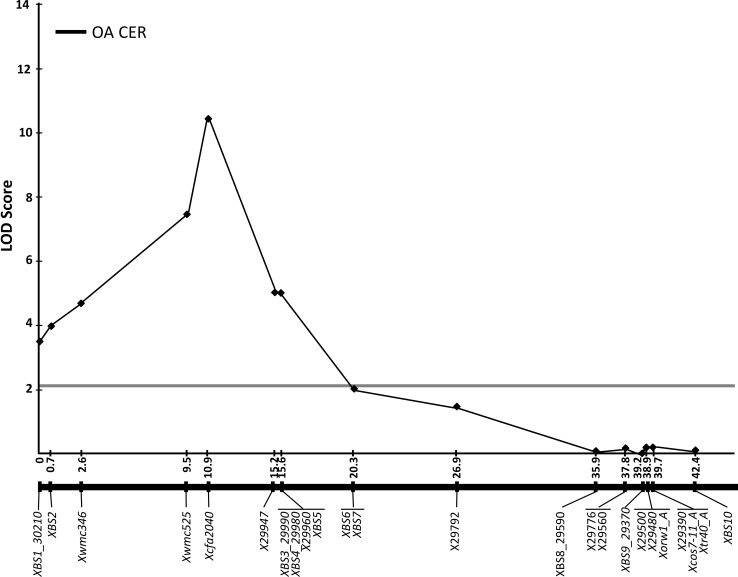
QTL interval mapping analysis of *Pch2* resistance to *Oculimacula acuformis* of the 92 wheat CS×CS/CD7A 7A F7 lines. This LOD profile derives from combined data of two CER trials. Genetic map data are aligned to the LOD profile

An interval mapping QTL analysis was carried out combining marker data with phenotypic results, and it revealed that the *Pch2* QTL peaks at *Xcfa2040* in the two bioassays explaining 26.1 and 33.5 % of the phenotypic variance observed in the first and second tests, respectively (Table 3). The combined data from the two trials also revealed that *Xcfa2040* (LOD = 10.41) was the marker most significantly associated with resistance (*Pch2*) explaining 42.4 % of the phenotypic variance observed. Overall, the analysis determined that the *Pch2* QTL region spans 15.6 cM in the interval between *XBS1_30210* and the co-segregating group of markers *XBS3_29990*-*XBS4_29980*-*X29960*-*XBS5* (Fig. 3; Table 3).

**Table 3:** Summary of the QTL interval mapping analysis of *Pch2* on chromosome 7AL

Locus	Genetic position	Trial 1 LOD^a^ score	Trial 2 LOD^a^ score	Combined data LOD^a^ score
*XBS1_30210*	0	2.35	3.54	3.54
*XBS2*	0.7	2.63	3.79	3.99
*Xwmc346*	2.7	4.02	3.9	4.7
*Xwmc525*	9.6	6.18	5.54	7.46
*Xcfa2040*	10.9	7.42	7.32	10.41
*X29947*	15.2	5.92	4.4	5.03
*X29960*	15.6	5.76	4.39	5.01
*XBS4_29980*	15.6	5.76	4.39	5.01
*XBS3_29990*	15.6	5.76	4.39	5.01
*XBS5*	15.6	5.76	4.39	5.01
*XBS7*	20.3	3.08	–	–
*XBS6*	20.3	3.08	–	–
*X29792*	26.9	2.79	–	–
	QTL effect:	*Xcfa2040*	*Xcfa2040*	*Xcfa2040*
	% Expl. Var:	26.1	33.5	42.4

^a^LOD score = −Log10(P). Significance threshold is 2.079

### *Pch1*–*Pch2* homoeology

**Fig. 4:**
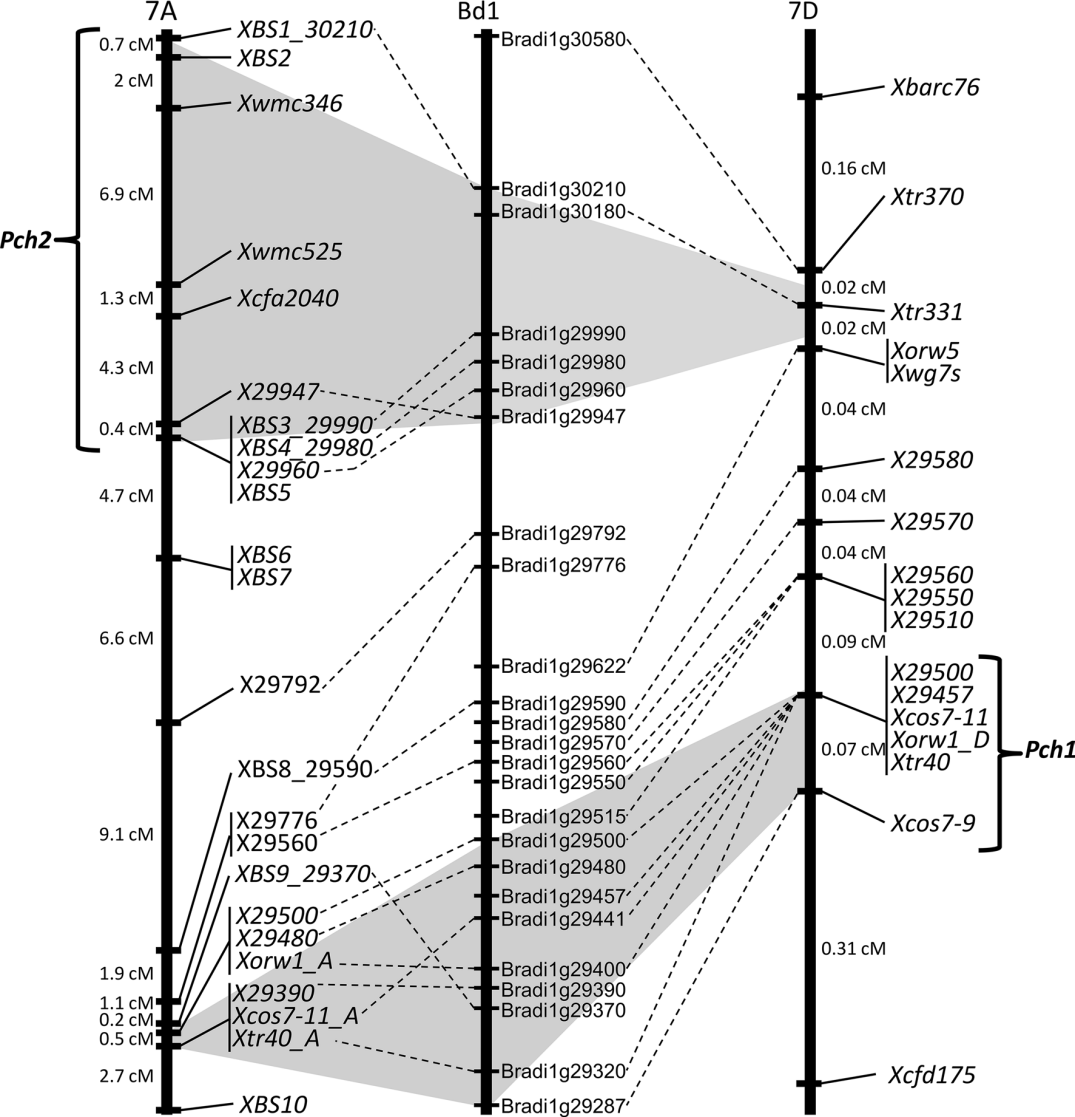
Comparison of the location of *Pch1* on the HSxRVPM25 genetic map of chromosome 7D (Ta7D) with the CSxCS/CD7A chromosome 7A genetic of *Pch2* using physical marker locations on Brachypodium chromosome 1 (Bd1) as a reference

The new genetic maps of 7D and 7A with the respective positions of *Pch1* and *Pch2* were then used to examine the potential homoeology between the two loci. The two maps were aligned to the relevant region of chromosome 1 of Bd on the basis of the overall syntenic relationship with Bd. Where possible, marker positions on both 7A and 7D chromosomes were anchored to the Bd gene-spaced map of the region (Fig. 4), and co-linearity was checked for both chromosomes against Bd1. Overall co-linearity was conserved across the entire length of the *Pch1* map. However, it should be noted that this map is that for the wheat-*Ae. ventricosa* population, and that due to the low level of recombination between wheat and *Ae. ventricosa*, it was not possible to dissect and confirm the gene order in the regions with the two co-segregating groups of markers mentioned above. Similarly, co-linearity was largely conserved between wheat 7A and Bd1 across the region analysed. However, a breakdown of the micro synteny was identified in the *Pch2* map, where *X29447*, *X29776*, *X29370*, and *Xcos7*-*11_A* seem to be located in a different position in respect to the physical order in Bd1 (Fig. 4). Using Bd1 as reference, the homoeologous region to *Pch1* on 7D was identified on chromosome 7A. The equivalent locus was located in a genetic region of 0.5 cM between the two co-segregating groups of markers *X29500*-*X29480*-*Xorw1_A* and *X29390*-*Xcos7*-*11_A*-*Xtr40_A*. The genetic distance between the most proximal of these groups (*X29500*-*X29480*-*Xorw1_A*) and the most distal group of markers (*XBS3_29990*-*XBS4_29980*-*X29960*-*XBS5*) for the *Pch2* QTL was 23.6 cM. On the basis of these new findings, it is clear that the *Pch1* and *Pch2* respective regions on chromosomes 7D and 7A do not overlap, and hence, there is no evidence that they represent homoeoloci.

### Molecular characterization of *T. durum* wheat lines carrying *Pch1* on 7A

Huguet-Robert et al. (2001) produced meiotically stable lines carrying *Pch1* on chromosome 7A in a background of the tetraploid *T. durum* variety Creso. Initially, a hybrid was obtained by crossing *Ae. ventricosa* accession 11 (Vent 11) containing *Pch1* on the 7D^v^ chromosome and *T. durum* cv. Creso *ph1c* mutant. The *Ph1* gene suppresses homoeologous pairing in wheat (Sears and Okamoto 1958), and mutations of this gene (*ph1b* and *ph1c*) were used to increase the chance of transferring *Pch1* containing 7D^v^ segment to wheat 7A or 7B chromosomes by homoeologous recombination. The hybrid was colchicine doubled and then put through a series of backcrossing to Creso *ph1c* and the hexaploid wheat variety Courtot *ph1b* with the aim of reducing and stabilising the chromosome number. Five homozygous eyespot resistant lines containing either 28 or 29 chromosomes were selected from this cross. The *Pch1* transfer in these lines was deduced to be located to the long arm of chromosome 7A, based on the apparent loss of the 7A specific SSR marker *Xgwm698* and the Ep-A1 protein. This suggested that a recombination event had occurred between 7A and 7D^v^ and so eliminating the region of 7A harbouring these loci. Some of these lines were used to produce F_3_ families in a backcross to Creso *ph1c* to further reduce the size of the *Pch1* introgression, and two homozygous eyespot resistant lines derived from line 301 (Huguet-Robert et al. 2001), namely, Red1 and Red5, were selected as potentially containing relatively small introgressions.

**Fig. 5:**
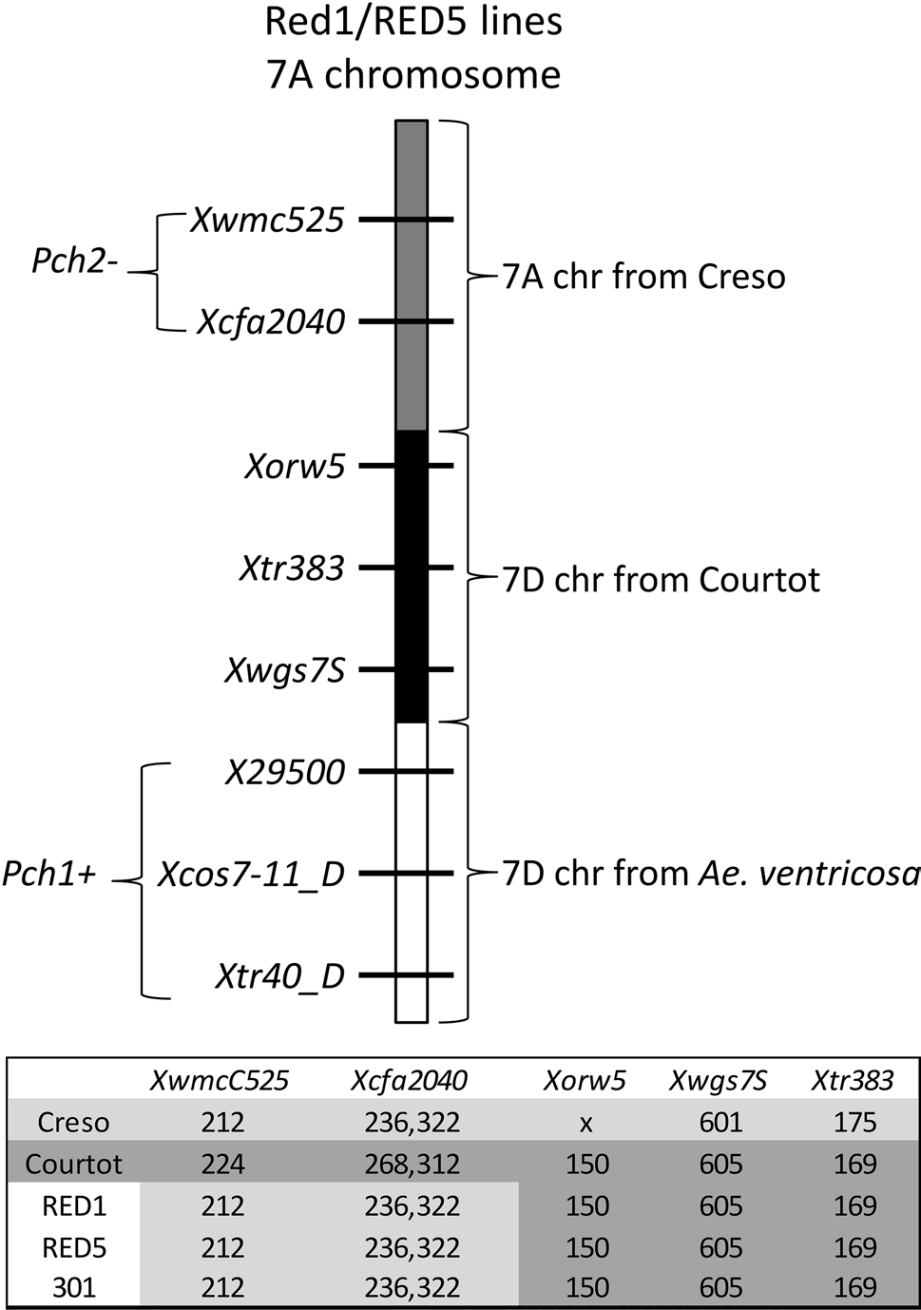
Chimeric arrangement of 7A chromosome of Red1 and Red 5 lines. Results from three 7D specific KASPs located inside the *Pch1* locus (*X29500*, *Xtr40_D*, and *Xcos7*-*11_D*) confirm that the *Pch1* locus was transferred to the end of 7A chromosome. Fragment size detected for the 7D specific SSRs *Xorw5*, *Xwgs7S*, and *Xtr383* and for the two 7A specific SSRs *Xwmc525* and *Xcfa2040* showed patterns identical to Courtot and Creso, respectively

To better characterize the nature and size of the *Pch1* introgression on chromosome 7A of these two lines, they were assayed for the presence of three 7D specific KASPs located within the region of the *Pch1* locus (*X29500*, *Xtr40_D*, and *Xcos7*-*11_D*). The three KASP assays revealed that Red1 and Red5 contained *Ae. ventricosa* alleles, so confirming that the *Pch1* locus had been transferred to the end of 7A chromosome. In an attempt to establish the extent of the *Ae. ventricosa* introgression and determine whether it extended into the region of *Pch2*, two 7A specific SSRs (*Xwmc525* and *Xcfa2040*) were run on the parental lines (Creso *ph1c*, Courtot *ph1b*, and *Ae. ventricosa* 11) and the two recombinant lines Red1 and Red5. Capillary electrophoresis of the *Xwmc525* and *Xcfa2040* PCR products revealed that they were identical to those of Creso, the *T. durum* recurrent parent (Fig. 5). This evidence indicates that the 7D^v^ introgression on 7A does not extend into the region, where *Pch2* is located. In the original report, evidence was provided that a portion of the Courtot hexaploid wheat parent was also transferred to the 7A chromosome of the *T. durum* (Huguet-Robert et al. (2001). The analysis of the lines using three 7D specific SSRs (*Xorw5*, *Xwgs7S*, and *Xtr383*) SSRs revealed that the product size detected in Red1 and Red5 was identical to that of Courtot (Fig. 5). These findings confirm the chimaeric nature of the arrangement of the 7A chromosome previously found by Huguet-Robert et al. (2001), and it is schematically represented in Fig. 5.

## Discussion

The main objective of this work was to refine the position of *Pch1* and *Pch2* on wheat chromosome 7D and 7A, respectively, to determine whether they are homoeoloci. The evidence generated by the present study clearly indicates that the two genes are not homoeoloci, and are genetically independent.

The *Pch1* resistance gene was introgressed to wheat from *Ae. ventricosa*. The cloning of this gene has been hampered by two main factors: marker availability and recombination rate. To deal with these issues, we exploited the syntenic relationship between Brachypodium and wheat for identifying a list of Conserved Orthologous Sequence (COS) markers located inside the *Pch1* region on chromosome 7D. These genes were used to tag interspecific (ventricosa-wheat) SNPs on which KASP markers were developed. These markers were then applied to the HS × RVPM25 BC_6_F_2_ population to discover new recombinants, and consequently, *Pch1* was located to a very small genetic region spanning only 0.07 cM between *Xcos7*-*9_D* and the group of co-segregating markers *X29500*-*X29457*-*Xcos7*-*11_D*-*Xorw1_D*-*Xtr40_D* (Fig. 1). Despite the larger number of lines screened (4512 gametes), a total of only 36 recombination events were identified between the two most external markers *Xbarc76* and *Xcdf175*, corresponding to recombination rate of 0.8 %. Considering that the rate of recombination between homoeologous chromosomes of wheat and wild grass relatives is estimated at around 3 % (Niu et al. 2011), our results suggest that the actual *Pch1* physical region is very small and that a recombination event within the introgressed segment is very unlikely to be observed. Further confirmation of this hypothesis is that the *Pch1*-corresponding genetic region mapped on the chromosome 7A in the cross CS × CS/CD7A (wheat × wheat), in which the recombination rate should be normal, spans only 0.5 cM (Fig. 4). Finally, in respect to the previous version of the map published by Burt and Nicholson (2011), the refined position of *Pch1* on the 7D eliminates 19 genes of the orthologous region on Bd1 (Fig. 1), which now physically spans only 250 Kb and contains 24 annotated genes. Taken together, these results are encouraging with respect to efforts towards cloning the *Pch1* gene, but indicate that different approaches have to be considered to overcome the biological (lack of recombination) and technical (marker saturation) limitations. The conventional approach would involve obtaining the physical sequence spanning the genetic interval through the screening of *ad hoc* created BAC libraries of the resistant genotype to identify a short list of candidates and test their nature by complementation. However, with the advent of the next-generation sequencing technologies, powerful alternatives to this conventional approach include mutagenesis, successfully used for cloning single-copy disease resistance genes in wheat (Feuillet et al. 2003; Periyannan et al. 2013), or exome capture methodologies and sequencing (Gardiner et al. 2014; Henry et al. 2014), which facilitate the cloning of genes from wheat and its relatives.

To refine the position of *Pch2*, we exploited the syntenic relationship between wheat and Brachypodium together with the high-density SNP genotyping 90 K array iSelect (Wang et al. 2014) to develop a new set of 20 KASP markers. The QTL analysis performed combining marker and phenotypic data resulted in positioning *Pch2* in a genetic region of 15.6 cM between *XBS1_30210* and the co-segregating group of markers *XBS3_29990*-*XBS4_29980*-*X29960*-*XBS5* (Fig. 3). Although this work used an increased number of markers combined with phenotyping of F_7_ lines, the genetic size of the interval containing *Pch2* remains unchanged in comparison with the previous version published by Burt and Nicholson (2011). Possible explanations for this could be the moderate phenotypic effect of *Pch2* (Burt et al. 2010; Chapman et al. 2008), which makes it difficult to monitor small differences in susceptibility using a visual disease scoring method based on leaf sheath penetration (Scott 1971) as well as the small size of the population (97 lines) used to perform the QTL analysis. However, the distal edge of the *Pch2* locus moved from the *Xcos7*-*11_A* marker to the group of co-segregating markers *XBS3_29990*-*XBS4_29980*-*X29960*-*XBS5*, which sits 24.1 cM away from *Xcos7*-*11_A* (Fig. 2). Moreover, using Brachypodium as a syntenic reference, this distance (from *Xcos7*-*11_A* corresponding to *Bradi1g29441* to *X29960* corresponding to *Bradi1g29960*) corresponds to a region containing 56 genes on Bd1 (Fig. 2).

In this work, two KASP markers, *Xorw1_A* and *Xorw1_D*, were designed to the 7A (Traes_7AL_D1DB1B9EE) and 7D (Traes_7DL_DBBA5FD6C) orthologous genes of *Bradi1g29400*. *Xorw1_D* is the KASP replacement of the STS marker (*Xorw1*) developed by (Leonard et al. 2008) towards the wheat EST AB246917. AB246917 is 96 and 91 % similar to Traes_7DL_DBBA5FD6C and *Bradi29400*, respectively, and encodes the endopeptidase protein Ep-D1b. A number of the previous studies detected a complete linkage between *Pch1* and either the isozyme marker (McMillin et al. 1986; Santra et al. 2006) or the STS marker (Burt and Nicholson 2011; Leonard et al. 2008; Meyer et al. 2011). In accordance with these studies, our data also did not identify any recombination event between *Xorw1_D* and *Pch1*, not making it possible to refute the hypothesis that Ep-D1b could be a candidate for *Pch1*. However, Mena et al. (1992) reported recombination between *Pch1* and allele *Ep*-*D1b* leading to the production of a line susceptible to eyespot but containing the *Ep*-*D1b* allele. Moreover, Huguet-Robert et al. (2001) obtained durum wheat lines using a second *Ae. ventricosa* accession (Vent 11), which are resistant to eyespot but lack a functional version of the Ep-D1 endopeptidase. Taken together, these results suggest that, while *Ep*-*D1b* is very closely linked to *Pch1*, it is not, itself, responsible for the enhanced eyespot resistance.

Koebner and Martin (1990) reported the eyespot resistance on the chromosome 7A of Cappelle Desprez to be associated to the endopeptidase allele *Ep*-*A1b*, and the isozyme marker of Ep-A1b was located at the end of chromosome 7A by de la Peña et al. (1997). In this work, we developed a KASP marker (*Xorw1_A*) based on Traes_7AL_D1DB1B9EE. Several lines of evidence lead us to consider this gene to be a good candidate for the *Ep*-*A1b* gene: Traes_7AL_D1DB1B9EE encodes an oligopeptidase B domain; it is located at the end of chromosome 7A (181.240 genetic bin); and it is the orthologous gene of *Bradi29400*, positioned in the *Pch1*/*Pch2* syntenic region of Bd1. Thus, *Xorw1_A* can be considered the first DNA-based replacement of the isozyme marker for Ep-A1b. Our data revealed that the recombination between *Pch2* and *Xorw1_A* was extensive with *Xorw1_A* being 28.1 cM from the *Pch2* peak marker (*Xcfa2040*) (Fig. 2). These results confirm the findings of de la Peña et al. (1997) who mapped the isozyme marker for Ep-A1b 32.8 cM distal to *Pch2*. However, the genetic distance of *Xorw1_A* from the QTL peak suggests that this marker is not suitable for the selection of *Pch2* resistance in wheat breeding.

Several papers have postulated about the possible homoelogy of *Pch1* and *Pch2*. Burt and Nicholson (2011) found that the region containing *Pch1* on chromosome 7D overlapped with the *Pch2* QTL on the chromosome 7A. Moreover, Chapman et al. (2008) identified an SSR marker *Xcfa2040* that is close to *Pch2* on the 7A, and also to the *Pch1*-linked SSR marker *Xwmc14* on the 7D.

The data from the present study do not support the view that *Pch1* and *Pch2* are homoeologous. In fact, we found that the homoeologous region to *Pch1* on chromosome 7A is located 23.6 cM away from the group of co-segregating markers (*XBS3_29990*-*XBS4_29980*-*X29960*-*XBS5*) that define the distal end of the *Pch2* QTL (Fig. 4). The most probable explanation for these discordant results is the difference in the resolution of genotyping and phenotyping of the plant material. We performed the eyespot resistance test on F_7_ families from the CS × CS/CD7A cross, minimising the influence of heterozygosity on phenotyping the moderately potent effect of *Pch2*. Moreover, adding a total of 11 and 21 new markers, respectively, to the *Pch1* and *Pch2* genetic maps allowed us to refine their positions on the respective chromosomes making a clear separation between them. In conclusion, our results reveal that *Pch1* and *Pch2* do not overlap and that they do not appear to be homoeoloci.

Finally, in this study, we have characterized Red1 and Red5 lines carrying *Pch1* on chromosome 7A in a background of the tetraploid *T. durum* variety Creso produced by Huguet-Robert et al. (2001).

Our aim was to define the nature and size of the *Pch1* introgression on chromosome 7A of these two lines and determine whether it extended into the region of *Pch2*. Marker analysis performed using three 7D specific KASPs located within the *Pch1* locus (*X29500*, *Xtr40_D*, and *Xcos7*-*11_D*) and two 7A specific SSRs (*Xwmc525* and *Xcfa2040*) located within the *Pch2* locus confirmed that Red1 and Red5 both contained the *Pch1* locus which had been transferred to the end of 7A chromosome. In addition, both the lines were identical to Creso, the *T. durum* recurrent parent in the *Pch2* region. Thus, the *Ae. ventricosa* introgression in these lines does not extend into the region of the 7A chromosome, where *Pch2* is located, making Red1 and Red5 potentially very interesting materials for future breeding programs to improve eyespot resistance. For example, it would be possible to cross these lines to a hexaploid wheat line containing *Pch2* and take advantage of recombination between 7A chromosomes from tetraploid and hexaploid wheat, to pyramid *Pch1*and *Pch2* in a tetraploid background. In addition, based on the finding that plants homozygous for *Pch1* are significantly more resistant than those that are heterozygous (Huguet-Robert et al. 2001), Red1 and Red5 can be used to create hexaploid genotypes with an increased copy number of *Pch1*, with one copy on 7D and one on 7A. Furthermore, it would be possible to cross them to *Pch1* containing lines, such as Rendezvous or Lynx, which also contain *Pch2* to produce lines containing both *Pch2* and one copy of *Pch1* on the chromosome 7A and one copy of *Pch1* on the 7D.

In conclusion, we provide evidence that does not support the view that *Pch1* and *Pch2* are homoeoloci. Consequently, this result has implications for the cloning of *Pch1*, because it prevents *Pch2* being used as a proxy target in which the higher level of recombination in wheat × wheat crosses could be exploited to fine map and identify the causal gene. On the other hand, the high level of wheat-Brachypodium synteny has been proven to be extremely helpful for guiding the development of new molecular markers and the identification of candidate genes, as a means to towards the cloning of *Pch1*. At the same time, the refined positions of *Pch1* and *Pch2* allowed us to characterize some potentially interesting material for pyramiding and/or increasing the copy number of different sources of eyespot resistance.

### Author contribution statement

MP, PN, CB, and CU made substantial contributions to the conception and design of the work. MP and JH carried out the acquisition and analysis of data and together with PN, CB, and JJ performed interpretation of data. JJ, SP, and CB contributed to the germplasm development used in the experiments. MP and PN wrote the paper. All the authors participated in revising it critically and gave approval of the final version to be submitted.
